# Optical Detection of Preneoplastic Lesions of the Central Airways

**DOI:** 10.5402/2012/957835

**Published:** 2012-03-22

**Authors:** C. van der Leest, A. Amelink, R. J. van Klaveren, H. C. Hoogsteden, H. J. C. M. Sterenborg, J. G. J. V. Aerts

**Affiliations:** ^1^Department of Respiratory Diseases, Erasmus Medical Center, Postbus 2040, 3000 CA Rotterdam, The Netherlands; ^2^Department of Respiratory Diseases, Amphia Hospital, Molengracht 21, 4818 CK Breda, The Netherlands; ^3^Center for Optical Diagnostics and Therapy and Department of Radiation Oncology, Erasmus Medical Center, Dr. Molewaterplein 50, 3015 GE Rotterdam, The Netherlands

## Abstract

Current routine diagnosis of premalignant lesions of the central airways is hampered due to a limited sensitivity (white light bronchoscopy) and resolution (computer tomography (CT), positron emission tomography (PET)) of currently used techniques. To improve the detection of these subtle mucosal abnormalities, novel optical imaging bronchoscopic techniques have been developed over the past decade. In this review we highlight the technological developments in the field of endoscopic imaging, and describe their advantages and disadvantages in clinical use.

## 1. Introduction

Lung cancer is the second most common cancer in men and women and the leading cause of cancer related death. In industrialized countries, the mortality rate of lung cancer is higher than that of breast, colorectal, and prostate cancer combined [1]. Lung cancer is divided in small cell lung cancer (SCLC) and non-small-cell lung cancer (NSCLC). NSCLC is subdivided in 3 different major histological classes: squamous cell carcinoma (SCC), adenocarcinoma, and undifferentiated large cell carcinoma [2]. Fifty years ago Auerbach et al. discovered that preinvasive lesions of different grades of severity were associated with lung tumors of squamous cell histology. This observation led to the hypothesis that SCC arises from these preinvasive changes [3]. It was shown that SCC develops sequentially: from normal to metaplasia, dysplasia, carcinoma *in situ* (CIS), and eventually invasive carcinoma [4]. Early detection of preinvasive lesions is important because local treatment can preclude patients from getting invasive cancers. Local treatment have been developed and includes photodynamic therapy (PDT) [5–7], electrocautery [8], brachytherapy [9], and cryotherapy [10]. Treatment with PDT has extensively been investigated. PDT showed in 99 patients with stage 0 and 56 patients with stage IA disease a complete response of 86%. Especially success response was seen in lesions smaller than 1 cm (complete response 95%) [11].

Since the epithelial changes associated with premalignancy are very subtle, no current routine imaging technique is sensitive enough to detect these lesions. Nonoptical imaging techniques such as ultrasound, magnetic resonance imaging (MRI), computer tomography (CT), and positron emission tomography (PET) do not have a sufficient spatial resolution to detect the subtle mucosal abnormalities. Currently, premalignant lesions are only detected by bronchoscopy.

Unfortunately, the sensitivity and specificity for the detection of premalignant lesions are low using standard white light bronchoscopy [12]. Therefore, novel endoscopic imaging techniques have been developed over the past decade to increase its sensitivity. Furthermore, optical point (spectroscopic) techniques have been developed to increase the specificity of the imaging modalities. In this paper we describe the technical aspects of these imaging and point measurement techniques, discuss the underlying biological mechanisms resulting in the optical contrast for each technique, and discuss the clinical use of these novel optical techniques.

## 2. Biological Changes

### 2.1. Morphology

SCC is mainly located in the central bronchial tree of the lung. This has been associated with cigarette smoke exposure and the higher concentration of carcinogens in the more central airways. The lesions are mainly located on the bifurcation segment bronchi, but no predominant place within these central airways is present. Comparable to the development of malignant lesions in other organs like esophageal cancer by chronic inflammation due to bile acid irritation and cervical cancer by chronic human papillomavirus (HPV) inflammation [13], lung cancer development seems to be driven by chronic irritation mostly due to not only smoking [14, 15] but also HPV inflammation [16]. As result of chronic irritation/stimulation, cells may differentiate towards a phenotype better adapted to the prevailing environment, and squamous metaplasia occurs [17].

It is believed that SCC develops according a stepwise process which can be observed with histological biopsies [4]. Hyperplasia and metaplasia are thought to be reactive lesions, with goblet cell hyperplasia and basal cell hyperplasia. Dysplasia is considered as a true preneoplastic lesion and may vary in degree, from mild to moderate and severe. Mild dysplastic lesions are characterized by minimal architectural and cytological disturbance. Moderate dysplasia exhibits more cytological irregularity, which is even more pronounced in severe dysplasia. In severe dysplasia it is accompanied by cellular polymorphism. In a subset of dysplastic changes, angiogenetic squamous dysplasia (ASD), the basal membrane thickens and there is vascular growth in the subepithelial tissues that results in papillary protrusions [18, 19]. The observed cellular changes include modifications in the volume, form, and orientation of the nuclei, an increase in nucleus chromatin content, variations in the nucleus-cytoplasm ratio, and changes in the intracytosolic content [20]. Also major architectural changes occur, such as a disorganized fibered network microstructure [21] and reticular basement membrane thickening [22], inducing a thickening of the epithelial layer.

### 2.2. Physiology

In several types of solid cancers, hypoxia has been reported as a key factor in the aggressiveness of a tumor [2, 23]. Heterogeneous distribution of oxygen and nutrients within the tumor has been related to invasive growth [24]. Some evidence exists that hypoxia is an early step in carcinogenesis [22, 25, 26], but for early lung cancer this has not been investigated. We did observe hypoxia in later stages of endobronchial cancer, but this cannot be extrapolated to premalignant disease [27].

Oxygenation of solid tumors is facilitated by the creation of new blood vessels (neoangiogenesis). In response to hypoxia, tumors secrete angiogenic cytokines, such as vascular endothelial growth factor (VEGF), inducing the formation of microvessels from the surrounding host vasculature [28]. Some studies suggest that neoangiogenesis is present in preinvasive and early invasive bronchial lesions, indicating that angiogenesis is an early event in lung cancer carcinogenesis [18, 29, 30]. Fisseler-Eckhoff et al. found an increasing vessel count in the bronchial mucosa when progressing from inflammation to hyperplasia, metaplasia, moderate dysplasia, severe dysplasia, and carcinoma *f* [29]. Angiogenetic squamous dysplasia (ASD) was identified in dysplastic bronchial epithelium [18, 19] as well. Whether or not ASD is an indicator of progression to invasive carcinoma is undetermined [31]. Furthermore, whether an increase in vascularisation is related to progression of a lesion to malignancy and whether it is driven by hypoxia remain to be elucidated.

Recently, Meert et al. did not find significant differences in microvessel count between different stages of premalignant bronchus carcinoma. However, they did find an elevated expression of neoangiogenetic proteins in premalignant lesions [32]. Furthermore, the use of vessel density to characterize premalignant lesions is limited because other biological factors such as infections, or chronic obstructive pulmonary disease, may increase the vessel density as well. In conclusion, the vasculature appears to be altered in premalignant disease, but data are not uniform, and no differentiation between changes in vessel density due to premalignant disease or due to other biological causes can be made.

### 2.3. Biochemistry

Hypothetically, the cellular metabolic activity is elevated in premalignant lesions, leading to intracellular changes in the concentration of nicotinamide adenine dinucleotide (NADH) and/or nicotinamide adenine phosphor dinucleotide (NADPH), which are both fluorescent molecules. In cancerous bronchial cells, a decreased fluorescence intensity of NADH and NADPH has been reported [33, 34]. Of note, these studies were performed in vitro, and translating these results to an *in vivo* environment is difficult, as discussed in Section 3.

## 3. Detection of Premalignant Lesions: Wide-Field Optical Imaging

Based on the possible biological mechanisms involved in early carcinogenesis, different techniques have been developed to detect premalignant lesions with higher sensitivity and specificity than white light bronchoscopy. Detection of premalignant lesions is usually performed in a wide-field imaging mode due to the large size of the bronchial tissue area that has to be investigated. In the following section we will discuss the technical aspects of the imaging techniques, relate the technology to the biological aspects of lung carcinogenesis, and discuss the clinical use of the techniques.

### 3.1. Autofluorescence Imaging

Autofluorescence bronchoscopy is the most widely used and investigated technique in the detection of premalignant bronchus lesions. Several autofluorescence devices are available. The LIFE (lung imaging fluorescence endoscopy) is the first and best known autofluorescence bronchoscope [35–44], first published by Lam et al. [12]. Other devices such as the D-light autofluorescence [45–47], Pentax-SAFE 1000 [48], and its successor the Pentax 3000 [11, 49, 50] are similar in their use.

Autofluorescence imaging uses the natural fluorescence properties of the tissue itself; that is, no exogenous contrast agents have been applied. When a natural fluorophore is excited to a higher electronic state by absorption of a photon of an appropriate wavelength, the fluorophore may return to its ground electronic state by emission of a photon of a higher wavelength (fluorescence). When the bronchial surface is illuminated with blue light (wavelengths ranging from 400 to 450 nm), the fluorescent light of a higher wavelength (>450 nm, i.e., green and red) can be visualized using long-pass filters that block the excitation light but transmit the higher wavelength fluorescent light. Since autofluorescence imaging is performed in a wide-field noncontact imaging mode, the detected fluorescent photons originate from various depths of tissue. The detected fluorescence consists of contributions from fluorophores that are involved in cellular metabolic processes such as nicotinamide adenine dinucleotide (NADH) and flavins (FAD) or are associated with their structural matrix (keratin, elastin, and collagen). In a wide-field imaging mode, it is expected that the latter fluorophores are the dominant contributors to the signal. Furthermore, spectral distortion of the measured fluorescence arises due to the scattering properties of tissue and absorption of both the excitation light and emitted fluorescence by tissue chromophores (specifically, hemoglobin). As a consequence, the optical contrast between premalignant and normal bronchial tissue using autofluorescence imaging is based on a combination of the three different biological mechanisms (biochemistry, physiology, and morphology). First, the local concentrations of natural fluorophores involved in cellular metabolism such as NADH may change in premalignant tissues [51]. However, since the dominant source of detected fluorescence is the structural matrix, this is not very likely to yield large contrasts. Second, an increased blood supply in the adjacent lamina propria is likely to play a key role in the reduction of autofluorescence in premalignant tissues and thus in the generation of the autofluorescence contrast in bronchial tissues [52]. However, since other biological factors such as infections, or chronic obstructive pulmonary disease, may increase the blood supply as well, this contrast is not likely to be very specific. Third, epithelial thickening will cause less excitation light to reach the structural matrix, thereby also reducing the detected fluorescence. Based on these biological mechanisms, premalignant tissue is expected to show reduced autofluorescence, but this effect will not be very specific.

These considerations are generally confirmed in clinical studies. Autofluorescence bronchoscopy was shown to increase the sensitivity for detection of premalignant bronchial lesions, but is hampered by a low specificity [35–44, 46, 47]. Difficulties exist in distinguishing benign epithelial chances such as bronchitis and inflammation from precancerous lesions. Studies have demonstrated about two-thirds of the lesions to be false positive results after correlation with pathology [38, 53, 54].

A number of modified versions of autofluorescence imaging have been introduced, which rely on the same principles of autofluorescence imaging but introduce additional filter sets to enhance the sensitivity of the images to the mucosal blood supply. The autofluorescence imaging bronchovideoscope system (AFI, Olympus) displays a composite image integrating autofluorescence with filtered reflected green and red light; the reflected green light is sensitive to mucosal blood due to the high absorption of green light by blood, whereas the reflected red light is basically unaffected by the presence of blood [55]. In a study including 32 patients, sensitivity of detecting dysplasia by AFI was 96.7% and specificity was 83.3% [55]. Another study, including 31 patients, sensitivity of detecting severe dysplasia or worse with AFI was 94.7% and the specificity was 71.1% [56].

The Onco-LIFE device contains a white light and a fluorescence mode. In fluorescence mode, the device uses blue light (395–455 nm) and small amount of red light (675–720 nm) from a filtered mercury arc lamp for illumination. The camera captured and combines the fluorescence green light and the reflected red light and displays it on a normal color video monitor. The green fluorescence light will change in places with bronchial pathology; the red reflected light is not affected by tissue pathology. Illuminated normal bronchial epithelium by blue light fluoresces in green. When blue light is illuminated on abnormal bronchial epithelium, it transforms through different grades of dysplasia into a progressive decrease in green fluorescence due to increased epithelial thickness and vascularization making these abnormal areas appear red. Red light is less absorbed by hemoglobin and therefore less influenced by changes in vascularity associated with inflammation [57]. The red reflected light can therefore be a reference for different light intensities from changes in angle and distance of the bronchoscope to the bronchial surface. Combining the reflected and fluorescence images enhances the contrast of normal, premalignant, and malignant bronchial tissue [57]. It is possible to analyze the red/green (R/G) ratio in the central portion of the displayed image providing quantitative data. Combining the data of two medical centers, 738 individuals underwent a bronchoscopy with the Onco-LIFE device. The corresponding R/G ratio increased when the premalignant lesions became more malignant. Validated data showed that R/G ratio of 0.54 had a 85% sensitivity and a 80% specificity for the detection of high-grade and moderate dysplasia [58]. The concept of color fluorescence ratio is not device specific and can be integrated into any reflectance fluorescence imaging system.

Lee et al. introduced real time dual video and autofluorescence bronchoscopic imaging [59], allowing to display both video and autofluorescence bronchoscopic images of the target simultaneously. This hypothetically makes it easier to identify benign lesions such as bronchitis, although the biological mechanism behind this hypothesis is not clear. Dual-band imaging has been studied in only one study, reporting a sensitivity and specificity in detection of preneoplastic lesions of 0.86 and 0.94, respectively [59]. However, sample size in that study was relatively small; secondly, it was not a comparative study where individual imaging modalities (video and AF bronchoscopy) were assessed independently before dual imaging.

The recently introduced color fluorescence endoscopic system, PDS-2000, uses a color-intensified charge-coupled device (ICCD) [60] instead of a regular CCD, but the advantages of such a device are not clear. In a clinical study, sensitivity and specificity were 89.2% and 52.6%, respectively, while for white light bronchoscopy these numbers 54.1% and 77.7%, respectively. No studies comparing color fluorescence bronchoscopy and autofluorescence bronchoscopy have been performed.

### 3.2. Narrow Band Imaging

Shibuya et al. developed narrow band imaging (NBI) [31]. The NBI-system (Olympus Corp, Tokyo, Japan) is based upon a light source with sequential red-green-blue (RGB) illumination. White light from a xenon lamp is passed through a rotary RGB filter that separates the white light into the colors red, green, and blue, which are used to sequentially illuminate the mucosa. The red, green, and blue reflected light is detected separately by a monochromatic charged coupled device (CCD) placed at the tip of the endoscope, and the three images are integrated into a single-color image by the video processor [31]. In addition to the conventional RGB filters for white light endoscopy, the narrow band imaging system has special RGB filters of which the band-pass ranges have been narrowed from the standard 400–700 nm (B 400–500 nm, G 500–600 nm, and R 600–700 nm) to 400–590 nm (B1 400–430 nm, B2 420–470 nm, and G 560–590 nm) [61]. Also, the relative contribution of blue light (B1) has been increased. With this choice of filters, NBI enables enhanced visualization of the mucosal morphology because blue light allows for optimal superficial imaging due to its small penetration depth. Secondly, because narrow band imaging filter B1 covers the absorption maximum for hemoglobin, a detailed image of the superficial vessel structures can be obtained. The NBI filter can be manually enabled and disabled during endoscopy making it easy to switch between the standard mode and the NBI mode. The biological hypothesis driving the development of NBI is that the microvascular patterns of premalignant lesions are different than those of normal bronchial mucosa [31]. In contrast to autofluorescence imaging, which is predominantly sensitive to total mucosal blood volume, NBI is sensitive to microvascular patterns with high spatial resolution, making the image contrast complementary to autofluorescence imaging [62, 63]. Clinically, microvessels, vascular networks, and dotted vessels were observed with NBI, and histological biopsies of areas with dotted vessels showed premalignant lesions and ASD [64]. NBI detected five instances of dysplasia or cancer (23% of all included patients) that were not detected with normal with light bronchoscopy (*P* < 0.005).

## 4. Improving the Specificity of Imaging: Point (Spectroscopic) Techniques

Novel imaging modalities increase the sensitivity for detection of premalignant bronchial lesions, at the cost of a low specificity. Difficulties exist in distinguishing benign epithelial chances such as bronchitis and inflammation from precancerous lesions. To increase the specificity of the imaging modalities, additional optical methods have been developed to be used in conjunction with wide-field imaging. These techniques provide more detailed information about small tissue areas/volumes, information that may be used to classify a suspicious lesion as either premalignant or benign. Although all technologies discussed in the next section are point techniques, they are categorized here as point imaging or point spectroscopic techniques.

### 4.1. Point Imaging

#### 4.1.1. Fibered Confocal Fluorescence Microscopy

Fibered confocal fluorescence microscopy (FCFM) is a technique that can be used to image the microscopic structure of the bronchial wall [21]. It is based on the principle of confocal microscopy, which provides a clear, in-focus image of a thin section within a biological sample by a flexible fiber-optic miniprobe.

The 1 mm diameter fiberoptic probe, which can be introduced into the working channel of a flexible bronchoscope, produces images from a layer of 0 to 50 *μ*m in depth below the bronchial surface, with a lateral resolution of 5 *μ*m, and a field of view up to 600 *μ*m in diameter [21]. This ultrahigh magnification system provides the endoscopist a cross-sectional image of the bronchial mucosa epithelium, resulting in images similar to histology during bronchoscopy [65, 66]. Basement membrane and upper submucosa can be made visible with a nice quality. Thiberville et al. tested FCFM in twenty-nine high-risk patients for lung cancer underwent an autofluorescence bronchoscopy. A specific pattern of the bronchial wall microstructure could be observed in some precancerous conditions showing a disorganized fibered network. This was observed in one invasive cancer, three CIS, two mild and one moderate dysplastic, and three metaplastic lesions [21]. However, the absence of visualisation of epithelial cells resulted in a difficulty of true differentiation of the premalignant bronchial lesions [65]. By adding an exogenous nontoxic fluorophore agent, such as methylene blue, reproducibility imaging of the epithelial cells could be obtained [66]. Future studies have to show whether FCFM with methylene blue is possible to differentiate between normal, premalignant, and malignant bronchus lesions.

FCFM can be easily performed during a bronchoscopy under local anesthesia. The miniprobe can be guided through the working channel of the bronchoscope. Interpretation of FCFM images of premalignant lesions relies on the fluorescence properties of the imaged tissue. Because the obtained images are similar to histology, the scopist must know the characteristics of premalignant histology to draw conclusions.

#### 4.1.2. Optical Coherence Tomography

Optical coherence tomography (OCT) is an optical imaging modality that performs high-resolution, cross-sectional, subsurface tomographic imaging of the microstructure of tissues [67]. It has been used to image subsurface tissue morphology in several other fields [68–75].The physical principle of OCT is similar to that of B-mode ultrasound imaging. Instead of using sound waves, near-IR light is passed into the tissue, and, by detecting the reflected light as it interacts with tissue structures as a function of depth, a cross-sectional image is created through optical interferometry [76]. In principle, OCT is capable of imaging the morphology of mucosal lesions. OCT is possible with an OCT probe with an outer diameter of 1.5 mm and a depth focus of 3 mm. It is possible to insert it in a working channel of a bronchoscope. For adequate subsurface tissue imaging, the OCT must be positioned closely to the airway wall. Images are real-time monitored.

Tsuboi et al. suggested that *in situ* and invasive carcinoma can be distinguished from normal bronchial epithelium [77]. Lam et al. concluded that autofluorescence endoscopy-guided OCT imaging of bronchial lesions is technically feasible and may be a promising nonbiopsy tool for *in vivo* imaging of preneoplastic bronchial lesions [78].

Interpretation of the OCT imaging is complex, and experience is required. To analyze the images and to differentiate between inflammatory and premalignant lesions, it is necessary to know more about the histopathology images of premalignant lesions. It seems that differentiation between different premalignant stages can be quantified by measurement of the epithelial thickness [78]. The epithelial thickness was significantly different between invasive cancer and CIS. Also the epithelial layer of mild, moderate, and severe dysplasia lesions was significantly thicker than of metaplastic lesions. 

The detection rate of premalignant lesions by OCT is probably related to the skills of the bronchoscopist and the individual imaging interpretation. Unfortunately the availability of OCT is limited due to the limitations of the current research results and the high costs of the equipment.

#### 4.1.3. Endocytoscopy System (ECS)

A recently introduced endoscopic microscopic technique is the endocytoscopy system (ECS) that enables microscopic imaging of the tracheobronchial tree [79]. ECS is able to magnify up to x450 on a video monitor. By staining areas of interest with methylene blue, high magnification imaging was possible. Images corresponded with the light microscopy. With this technique *in vivo* discrimination between normal and dysplastic lesions is possible. A limitation of the technique is that contact with the bronchial tree is necessary, which may cause bleeding, hampering a clear view. Unfortunately, no clinical data are available at the moment.

### 4.2. Point Spectroscopy

#### 4.2.1. Autofluorescence Spectroscopy

Autofluorescence spectroscopy works similar to autofluorescence imaging, but instead of visualising the remitted fluorescence of a large tissue area using a CCD camera with only a few wavelength bands (mostly red and/or green), the remitted fluorescence of a small tissue area is coupled (usually with a fiber) into a spectrometer which resolves the fluorescence into a complete spectrum [80]. However, AFS suffers from the same problems as autofluorescence imaging: it cannot discriminate between premalignant and benign disease such as bronchitis and inflammation [80].

#### 4.2.2. Reflectance Spectroscopy

The absorption and scattering coefficients of tissue are wavelength dependent, and their value at each wavelength reflects the probability that a photon will be absorbed or scattered by the tissue. The absorption coefficient is given by the product of the extinction coefficient and the concentration of dominant tissue chromophores such as oxygenated hemoglobin (HbO_2_) and deoxygenated hemoglobin (Hb), bilirubin, beta-carotene, water, and lipids. The absorption coefficient is therefore also related to physiological parameters such as total hemoglobin concentration (THb = HbO_2_ + Hb) and blood oxygen saturation (StO_2_ = HbO_2_/THb). Scattering in tissue originates from spatial heterogeneities of the optical refractive index that occur on size scales ranging from a few nanometers to a few millimeters. Since these refractive index fluctuations depend on the concentration and type of tissue constituents, the light-scattering signature (both the light-scattering amplitude and wavelength dependence) is sensitive to the microarchitecture of the tissue and can be used for tissue diagnosis. Differential path length spectroscopy (DPS) is a novel reflectance spectroscopic technique developed for the purpose of studying the superficial layer of the bronchial mucosa [27, 81–83]. DPS utilizes a unique fiber-optic geometry to selectively sample photons that have propagated shallow depths into tissue, making it very suitable for the classification of superficial lesions such as preneoplasias. In DPS the path length is approximately equal to the fiber diameter, independent of absorption and scattering. This fiber geometry overcomes the classical limitation of unknown photon path length during diffuse reflectance measurements; this allows real-time quantitative information (THb, StO2) to be extracted from DPS measurements. Using DPS we have measured a significant difference in THb and StO2 between normal tissue and cancerous tissue. However, no significant differences in these parameters were found between normal and premalignant tissues [82], although the number of truly premalignant lesions (dysplasia, CIS) included in that study was very low.

Zeng et al. used an integrated endoscopy system for simultaneous imaging and spectroscopy. This system is capable of obtaining white-light bronchoscopy, autofluorescence bronchoscopy, and both reflectance and fluorescence spectroscopies without introducing optical fibers through the working channel [84]. The advantage of this technique is the noncontact procedure. The integrated endoscopy system was tested in 63 patients and showed that the experimental system was able to provide identical spectra to those obtained by fiber-optic probes [85]. Significant differences of reflectance and fluorescence spectra from malignant tissue compared to normal lung tissue were observed. Due to the limited numbers of premalignant lesions, no information of improving the sensitivity or specificity of detection of premalignant lesions could be presented.

## 5. Discussion

No single optical detection modality is sufficiently accurate to gain clinical acceptance as a screening tool for preneoplastic bronchial lesions. Except for autofluorescence bronchoscopy, limited patient data on these new techniques are available. Autofluorescence bronchoscopy has proven its value in the detection of neoplastic lesions, but unfortunately the low specificity limits this technique to be used as a screenings tool.

Based on the currently available techniques a combination of a wide-field optical imaging technique and a point (imaging) technique might improve the detection rate. A wide-field technique is used to locate several suspicious lesions, and a point imaging technique is subsequently used to differentiate between true positive or false positive lesions.

For the first purpose, autofluorescence bronchoscopy seems momentarily the best wide-field optical technique since a large number of investigations has been proven that this technique is with a high sensitivity.

For the second purpose, OCT combined with DPS has the highest potential value. OCT provides a histological-like diagnosis on site based on endogenous contrast, which increases the specificity of the fluorescence bronchoscopy during bronchoscopy, and it is the only noncontact point imaging technique. The noncontact possibility prevents contact bleeding and therefore misinterpretations of investigated lesions. Furthermore, it is a technique where quantitative data of the epithelial thickness can help to draw conclusions about the seriousness of a premalignant lesion.

DPS informs about the superficial morphology and physiology of the bronchial tissue and therefore provides information complementary to OCT images. The incorporation of these three techniques includes the three important conditions of detection of premalignant lesions: firstly, a wide-field technique for detection of suspicious lesions, secondly, a point imaging technique to give more local information about the histology of the suspicious lesions, and thirdly, a spectroscopic technique which gives information about the physiology of the local tissue.

## 6. Conclusion

In conclusion, a combination of autofluorescence bronchoscopy with OCT and DPS seems for now the best technique to be used in future studies on premalignant lesions. In addition, none of the presented techniques, or a combination of techniques, can be advised to be used outside a research setting.

## References

[1] Jemal A, Siegel R, Ward E, Murray T, Xu J, Thun MJ (2007). Cancer statistics, 2007. *Ca-A Cancer Journal for Clinicians*.

[2] Travis WD, Brambilla E, Muller-Hermelink HK, Harris CC (2004). *Pathology and genetics of Tumours of the Lung, Pleura, Thymus and Heart*.

[3] Auerbach O, Forman JB, Gere JB (1957). Changes in the bronchial epithelium in relation to smoking and cancer of the lung; a report of progress. *The New England Journal of Medicine*.

[4] Nettesheim P, Griesemer RA, Martin DH, Caton JE (1977). Induction of preneoplastic and neoplastic lesions in grafted rat tracheas continuously exposed to benzo(a)pyrene. *Cancer Research*.

[5] Ono R, Ikeda S, Suemasu K (1992). Hematoporphyrin derivative photodynamic therapy in roentgenographically occult carcinoma of the tracheobronchial tree. *Cancer*.

[6] Chiaia NL, Bennett-Clarke CA, Rhoades RW (1991). Effects of cortical and thalamic lesions upon primary afferent terminations, distributions of projection neurons, and the cytochrome oxidase pattern in the trigeminal brainstem complex. *Journal of Comparative Neurology*.

[7] Edell ES, Cortese DA (1987). Bronchoscopic phototherapy with hematoporphyrin derivative for treatment of localized bronchogenic carcinoma: a 5-year experience. *Mayo Clinic Proceedings*.

[8] van Boxem TJ, Venmans BJ, Schramel FM (1998). Radiographically occult lung cancer treated with fibreoptic bronchoscopic electrocautery: a pilot study of a simple and inexpensive technique. *European Respiratory Journal*.

[9] Pérol M, Caliandro R, Pommier P (1997). Curative irradiation of limited endobronchial carcinomas with high-dose rate brachytherapy: results of a pilot study. *Chest*.

[10] Deygas N, Froudarakis M, Ozenne G, Vergnon JM (2001). Cryotherapy in early superficial bronchogenic carcinoma. *Chest*.

[11] Kennedy TC, McWilliams A, Edell E (2007). Bronchial intraepithelial neoplasia/early central airways lung cancer: ACCP evidence-based clinical practice guidelines (2nd edition). *Chest*.

[12] Lam S, MacAulay C, Hung J, LeRiche J, Profio AE, Palcic B (1993). Detection of dysplasia and carcinoma in situ with a lung imaging fluorescence endoscope device. *Journal of Thoracic and Cardiovascular Surgery*.

[13] Sital RR, Kusters JG, de Rooij FW, Kuipers EJ, Siersema PD (2006). Bile acids and Barrett's oesophagus: a sine qua non or coincidence?. *Scandinavian Journal of Gastroenterology. Supplement*.

[14] Güngör N, Haegens A, Knaapen AM (2010). Lung inflammation is associated with reduced pulmonary nucleotide excision repair in vivo. *Mutagenesis*.

[15] Pauly JL, Smith LA, Rickert MH, Hutson A, Paszkiewicz GM (2010). Review: is lung inflammation associated with microbes and microbial toxins in cigarette tobacco smoke?. *Immunologic Research*.

[16] Syrjanen KJ (2002). HPV infections and lung cancer. *Journal of Clinical Pathology*.

[17] Kerr KM (2001). Pulmonary preinvasive neoplasia. *Journal of Clinical Pathology*.

[18] Keith RL, Miller YE, Gemmill RM (2000). Angiogenic squamous dysplasia in bronchi of individuals at high risk for lung cancer. *Clinical Cancer Research*.

[19] Franklin WA (2000). Diagnosis of lung cancer: pathology of invasive and preinvasive neoplasia. *Chest*.

[20] Brambilla E, Travis WD, Colby TV, Corrin B, Shimosato Y (2001). The new World Health Organization classification of lung tumours. *European Respiratory Journal*.

[21] Thiberville L, Moreno-Swirc S, Vercauteren T, Peltier E, Cavé C, Heckly GB (2007). In vivo imaging of the bronchial wall microstructure using fibered confocal fluorescence microscopy. *American Journal of Respiratory and Critical Care Medicine*.

[22] Polosukhin VV, Lawson WE, Milstone AP (2007). Association of progressive structural changes in the bronchial epithelium with subepithelial fibrous remodeling: a potential role for hypoxia. *Virchows Archiv*.

[23] Höckel M, Vaupel P (2001). Tumor hypoxia: definitions and current clinical, biologic, and molecular aspects. *Journal of the National Cancer Institute*.

[24] Frieboes HB, Zheng X, Sun CH, Tromberg B, Gatenby R, Cristini V (2006). An integrated computational/experimental model of tumor invasion. *Cancer Research*.

[25] Griffiths EA, Pritchard SA, McGrath SM (2007). Increasing expression of hypoxia-inducible proteins in the Barrett’s metaplasia-dysplasia-adenocarcinoma sequence. *British Journal of Cancer*.

[26] Griffiths EA, Pritchard SA, Valentine HR (2007). Hypoxia-inducible factor-1*α* expression in the gastric carcinogenesis sequence and its prognostic role in gastric and gastro-oesophageal adenocarcinomas. *British Journal of Cancer*.

[27] Bard MPL, Amelink A, Skurichina M (2006). Optical spectroscopy for the classification of malignant lesions of the bronchial tree. *Chest*.

[28] Ciardiello F (2005). Epidermal growth factor receptor inhibitors in cancer treatment. *Future Oncology*.

[29] Fisseler-Eckhoff A, Rothstein D, Müller KM (1996). Neavascularization in hyperplastic, metaplastic and potentially preneoplastic lesions of the bronchial mucosa. *Virchows Archiv*.

[30] Fontanini G, Calcinai A, Boldrini L (1999). Modulation of neoangiogenesis in bronchial preneoplastic lesions. *Oncology Reports*.

[31] Shibuya K, Hoshino H, Chiyo M (2003). High magnification bronchovideoscopy combined with narrow band imaging could detect capillary loops of angiogenic squamous dysplasia in heavy smokers at high risk for lung cancer. *Thorax*.

[32] Meert AP, Feoli F, Martin B, Ninane V, Sculier JP (2007). Angiogenesis in preinvasive, early invasive bronchial lesions and micropapillomatosis and correlation with EGFR expression. *Histopathology*.

[33] Pitts JD, Sloboda RD, Dragnev KH, Dmitrovsky E, Mycek MA (2001). Autofluorescence characteristics of immortalized and carcinogen-transformed human bronchial epithelial cells. *Journal of Biomedical Optics*.

[34] Wagnières GA, Star WM, Wilson BC (1998). In vivo fluorescence spectroscopy and imaging for oncological applications. *Photochemistry and Photobiology*.

[35] Lam S, Kennedy T, Unger M (1998). Localization of bronchial intraepithelial neoplastic lesions by fluorescence bronchoscopy. *Chest*.

[36] Shibuya K, Fujisawa T, Hoshino H (2001). Fluorescence bronchoscopy in the detection of preinvasive bronchial lesions in patients with sputum cytology suspicious or positive for malignancy. *Lung Cancer*.

[37] Sato M, Sakurada A, Sagawa M (2001). Diagnostic results before and after introduction of autofluorescence bronchoscopy in patients suspected of having lung cancer detected by sputum cytology in lung cancer mass screening. *Lung Cancer*.

[38] Hirsch FR, Prindiville SA, Miller YE (2001). Fluorescence versus white-light bronchoscopy for detection of preneoplastic lesions: a randomized study. *Journal of the National Cancer Institute*.

[39] Chhajed PN, Shibuya K, Hoshino H (2005). A comparison of video and autofluorescence bronchoscopy in patients at high risk of lung cancer. *European Respiratory Journal*.

[40] Kurie JM, Lee JS, Morice RC (1998). Autofluorescence bronchoscopy in the detection of squamous metaplasia and dysplasia in current and former smokers. *Journal of the National Cancer Institute*.

[41] Vermylen P, Pierard P, Roufosse C (1999). Detection of bronchial preneoplastic lesions and early lung cancer with fluorescence bronchoscopy: a study about its ambulatory feasibility under local anaesthesis. *Lung Cancer*.

[42] Kusunoki Y, Imamura F, Uda H, Mano M, Horai T (2000). Early detection of lung cancer with laser-induced fluorescence endoscopy and spectrofluorometry. *Chest*.

[43] van Rens MTM, Schramel FMNH, Elbers JRJ, Lammers JWJ (2001). The clinical value of lung imaging fluorescence endoscopy for detecting synchronous lung cancer. *Lung Cancer*.

[44] Ikeda N, Honda H, Katsumi T (1999). Early detection of bronchial lesions using lung imaging fluorescence endoscope. *Diagnostic and Therapeutic Endoscopy*.

[45] Haussinger K, Stanzel F, Markus A, Bolliger CT, Pichler J (1999). Early diagnosis of bronchial carcinoma: technical endoscopic progress—a step toward new screening concepts?. *Pneumologie*.

[46] Fuso L, Pagliari G, Boniello V (2005). Autofluorescence bronchoscopy to identify pre-cancerous bronchial lesions. *Monaldi Archives for Chest Disease*.

[47] Beamis JF, Ernst A, Simoff M, Yung R, Mathur P (2004). A multicenter study comparing autofluorescence bronchoscopy to white light bronchoscopy using a non-laser light stimulation system. *Chest*.

[48] Baletic N, Petrovic Z, Pendjer I, Malicevic H (2004). Autofluorescent diagnostics in laryngeal pathology. *European Archives of Oto-Rhino-Laryngology*.

[49] Ikeda N, Honda H, Hayashi A (2006). Early detection of bronchial lesions using newly developed videoendoscopy-based autofluorescence bronchoscopy. *Lung Cancer*.

[50] Usuda J, Tsutsui H, Honda H (2007). Photodynamic therapy for lung cancers based on novel photodynamic diagnosis using talaporfin sodium (NPe6) and autofluorescence bronchoscopy. *Lung Cancer*.

[51] Pogue BW, Pitts JD, Mycek MA (2001). In Vivo NADH fluorescence monitoring as an assay for cellular damage in photodynamic therapy. *Photochemistry and Photobiology*.

[52] Gabrecht T, Andrejevic-Blant S, Wagnieres G (2006). *Blue-Violet Excited Autofluorescence Spectroscopy and Imaging of Normal and Cancerous Human Bronchial Tissue after Formalin Fixation*.

[53] Banerjee AK, Rabbitts PH, George J (2003). Lung cancer • 3: fluorescence bronchoscopy: clinical dilemmas and research opportunities. *Thorax*.

[54] Kennedy TC, Lam S, Hirsch FR (2001). Review of recent advances in fluorescence bronchoscopy in early localization of central airway lung cancer. *Oncologist*.

[55] Chiyo M, Shibuya K, Hoshino H (2005). Effective detection of bronchial preinvasive lesions by a new autofluorescence imaging bronchovideoscope system. *Lung Cancer*.

[56] Ueno K, Kusunoki Y, Imamura F (2007). Clinical experience with autofluorescence imaging system in patients with lung cancers and precancerous lesions. *Respiration*.

[57] Edell E, Lam S, Pass H (2009). Detection and localization of intraepithelial neoplasia and invasive carcinoma using fluorescence-reflectance bronchoscopy: an international, multicenter clinical trial. *Journal of Thoracic Oncology*.

[58] Lee P, van den Berg RM, Lam S (2009). Color fluorescence ratio for detection of bronchial dysplasia and carcinoma in situ. *Clinical Cancer Research*.

[59] Lee P, Brokx HAP, Postmus PE, Sutedja TG (2007). Dual digital video-autofluorescence imaging for detection of pre-neoplastic lesions. *Lung Cancer*.

[60] Nakanishi K, Ohsaki Y, Kurihara M (2007). Color auto-fluorescence from cancer lesions: improved detection of central type lung cancer. *Lung Cancer*.

[61] Gono K, Obi T, Yamaguchi M (2004). Appearance of enhanced tissue features in narrow-band endoscopic imaging. *Journal of Biomedical Optics*.

[62] Votruba J, Bruha T, Javorsky S, Zavadil J, Kostka F (2007). Discriminating value of three bronchoscopic techniques—autofluorescence bronchoscopy, autofluorescence spectroscopy and narrow band imaging. *Journal of Thoracic Oncology*.

[63] Herth FJF, Eberhardt R, Anantham D, Gompelmann D, Zakaria MW, Ernst A (2009). Narrow-band imaging bronchoscopy increases the specificity of bronchoscopic early lung cancer detection. *Journal of Thoracic Oncology*.

[64] Vincent BD, Fraig M, Silvestri GA (2007). A pilot study of narrow-band imaging compared to white light bronchoscopy for evaluation of normal airways and premalignant and malignant airways disease. *Chest*.

[65] Thiberville L, Salaün M, Lachkar S (2009). Confocal fluorescence endomicroscopy of the human airways. *Proceedings of the American Thoracic Society*.

[66] Thiberville SM, Dominique S, Moreno-Swirc S, Vever-Bizet C, Bourg-Heckly G Confocal micro-endosocopy.

[67] Whiteman SC, Yang Y, van Pittius DG, Stephens M, Parmer J, Spiteri MA (2006). Optical coherence tomography: real-time imaging of bronchial airways microstructure and detection of inflammatory/neoplastic morphologic changes. *Clinical Cancer Research*.

[68] Sakata LM, Deleon-Ortega J, Sakata V, Girkin CA (2009). Optical coherence tomography of the retina and optic nerve—a review. *Clinical & Experimental Ophthalmology*.

[69] Mirza RG, Johnson MW, Jampol LM (2007). Optical coherence tomography use in evaluation of the vitreoretinal interface: a review. *Survey of Ophthalmology*.

[70] Gambichler T, Moussa G, Sand M, Sand D, Altmeyer P, Hoffmann K (2005). Applications of optical coherence tomography in dermatology. *Journal of Dermatological Science*.

[71] Farooq MU, Khasnis A, Majid A, Kassab MY (2009). The role of optical coherence tomography in vascular medicine. *Vascular Medicine*.

[72] Testoni PA, Mangiavillano B (2008). Optical coherence tomography in detection of dysplasia and cancer of the gastrointestinal tract and bilio-pancreatic ductal system. *World Journal of Gastroenterology*.

[73] Poneros JM (2004). Diagnosis of Barrett’s esophagus using optical coherence tomography. *Gastrointestinal Endoscopy Clinics of North America*.

[74] Karl A, Stepp H, Willmann E (2008). Optical coherence tomography (OCT): ready for the diagnosis of a nephrogenic adenoma of the urinary bladder?. *Journal of Endourology*.

[75] Schmidbauer J, Remzi M, Klatte T (2009). Fluorescence cystoscopy with high-resolution optical coherence tomography imaging as an adjunct reduces false-positive findings in the diagnosis of urothelial carcinoma of the bladder. *European Urology*.

[76] Sheppard DSaC www.obel.ee.uwa.edu.au/research.

[77] Tsuboi M, Hayashi A, Ikeda N (2005). Optical coherence tomography in the diagnosis of bronchial lesions. *Lung Cancer*.

[78] Lam S, Standish B, Baldwin C (2008). In vivo optical coherence tomography imaging of preinvasive bronchial lesions. *Clinical Cancer Research*.

[79] Shibuya KFT, Wada H, Nagato K (2009). *Novel Endoscopic Microscopy Using Endo-Cytoscopy System*.

[80] DaCosta RS, Wilson BC, Marcon NE (2007). Fluorescence and spectral imaging. *TheScientificWorldJournal*.

[81] Bard MPL, Amelink A, Skurichina M (2005). Improving the specificity of fluorescence bronchoscopy for the analysis of neoplastic lesions of the bronchial tree by combination with optical spectroscopy: preliminary communication. *Lung Cancer*.

[82] Bard MPL, Amelink A, Hegt VN (2005). Measurement of hypoxia-related parameters in bronchial mucosa by use of optical spectroscopy. *American Journal of Respiratory and Critical Care Medicine*.

[83] Aerts JGJV, Amelink A, van der Leest C (2007). HIF1a expression in bronchial biopsies correlates with tumor microvascular saturation determined using optical spectroscopy. *Lung Cancer*.

[84] Zeng H, Petek M, Zorman MT, McWilliams A, Palcic B, Lam S (2004). Integrated endoscopy system for simultaneous imaging and spectroscopy for early lung cancer detection. *Optics Letters*.

[85] Tercelj M, Zeng H, Petek M, Rott T, Palcic B (2005). Acquisition of fluorescence and reflectance spectra during routine bronchoscopy examinations using the ClearVu Elite*™* device: pilot study. *Lung Cancer*.

